# A database of mapped global fishing activity 1950–2017

**DOI:** 10.1038/s41597-023-02824-6

**Published:** 2024-01-08

**Authors:** Yannick Rousseau, Julia L. Blanchard, Camilla Novaglio, Kirsty A. Pinnell, Derek P. Tittensor, Reg A. Watson, Yimin Ye

**Affiliations:** 1 Institute for Marine and Antarctic Studies, University of Tasmania, Hobart, TAS Australia; 2 Department of Biology, Dalhousie University, Halifax, NS Canada; 3 Fisheries and Aquaculture Division, Food and Agriculture Organization of the United Nations, Rome, Italy

**Keywords:** Interdisciplinary studies, Ocean sciences, Environmental sciences, Environmental social sciences

## Abstract

A new database on historical country-level fishing fleet capacity and effort is described, derived from a range of publicly available sources that were harmonized, converted to fishing effort, and mapped to 30-min spatial cells. The resulting data is comparable with widely used but more temporally-limited satellite-sourced Automatic Identification System (AIS) datasets for large vessels, while also documenting important smaller fleets and artisanal segments. It ranges from 1950 to 2017, and includes information on number of vessels, engine power, gross tonnage, and nominal effort, categorized by vessel length, gear type and targeted functional groups. The data can be aggregated to Large Marine Ecosystem, region and/or fishing country scales and provides a temporally and spatially explicit source for fishing effort and fleet capacity for studies aimed at understanding the implications of long-term changes in fishing activity in the global ocean.

## Background & Summary

Marine fisheries have historically been and will continue to be a globally important source of nutrition, employment, and livelihood^1^. In recent years, there has been a push towards inclusion of fisheries-data in global ecological models in order to address the multiple challenges faced by marine ecosystems, communities, and the sector itself, such as climate change^2^, depletion of the oceans^3^, and regional dependence on the productivity of adjacent terrestrial systems^4^. Fisheries yields are ultimately limited by the size and productivity of the stocks and their interaction with the supporting marine ecosystems, with all these factors being spatially dependent. With the widespread nature of fishing and its impact on ecosystems, there is a need for a detailed understanding of fishing effort, geographical and historical patterns in particular^5,6^.

While the use of fishing capacity (number of vessels and characteristics) and effort (utilization of capacity) as parameters in ecosystem models is not new, that of global spatially disaggregated effort is more recent^7^. Recent advances in standardisation of telecommunication systems have further provided insights on the precise location of the vessels^8^. Vessel automatic identification systems (AIS) can be used to infer the location of fishing effort, and AIS has shown itself a promising instrument for tracking the effort of fishing fleets^8^. AIS data, however, is limited temporally to recent years, and in scope to industrial fleets and not all countries. Thus, there remains a need for long-term fishing effort data, both in terms of providing historical patterns and an alternative data source supplementing the inherent limitations of AIS data^9^.

While the wide-spread adoption of AIS might prove extremely useful for fisheries management, there is a need to understand the limitations of such data, if only to avoid falling into the ‘technology effect’ fallacy^10^, a tendency to overestimate the success of implementing new technologies. In particular, concerns have been raised over large-scale manipulation of AIS data and its links to corruption^11^, raising questions on the validity of the former. Furthermore, AIS data is heavily dependent on receiver coverage, leading to a heterogenous accuracy across the globe^8^. Comparing AIS with national-level fishing capacity data and catch-based methods for mapping fishing effort^7^ is a first step towards cross validation and integration.

In terms of longer-term historical data, previous maps of global fishing effort from national yearbooks have been reconstructed but have been biased towards certain regions^12^, with western industrial regions (North America, Europe) overrepresented relative to other ‘big players’ such as China, Canada, Indonesia, and fisheries of Small Island Nations often confused with the Tuna fisheries they host^12^ Here, we address those limitations by describing an updated global and capacity database^13^, disaggregated to gear, vessel length, and engine power categories, mapped to 30-min grid cells, based on country-level statistics obtained from national yearbooks and other publicly available sources. We describe the steps involved in the creation of the database, including the reconstruction of country-level effort time series and allocation to spatial grid cells globally^12^. For the latter, we assume the same heuristic rules holds for spatial mapping of country-level effort data as has been done previously for catch and effort^14–16^. We then compare the reconstructed mapped effort with AIS tracking data presented in the Global Fishing Watch database (globalfishingwatch.org) to verify the validity, gaps and biases of both methods, and direct future research.

The result is a series of databases of aggregated and mapped fishing activity, including number of vessels, engine power, gross tonnage, and nominal and effective effort in days at sea × kW, by gear type, functional group of targeted species and size of vessel (length) along a 30-min gridded map, as well as conversion factors to transform days at sea to fishing hours.

## Methods

**Fig. 1:**
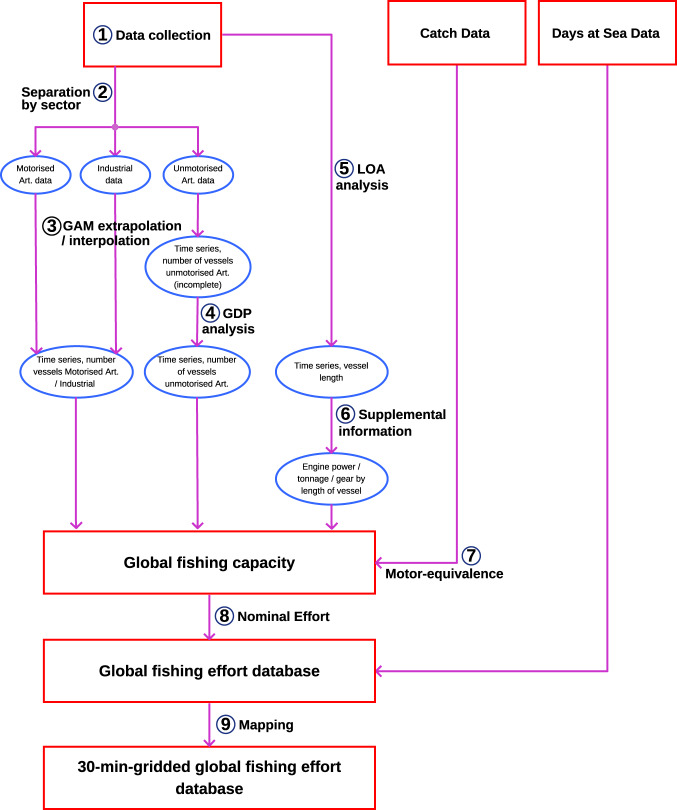
Processes used in estimating the global marine fishing capacity and effort. Supplementary processes are described in the corresponding sections. LOA – Length overall (of vessels). GAM – Generalized Additive Models. GDP – Gross Domestic Product.

Data were gathered from a range of publicly available sources, governmental reports, and grey literature. They were combined as time series of number of vessels, then separated in classes based on the vessel length, and then allocated to fishing gear type, main engine power, gross tonnage, and finally days at sea using previously published methods and encoding^12,17^. The data was associated with currently published catch datasets^16^ to allow mapping of the effort along 30 min spatial cells (Fig. 1). [Further information on each step can be found in the Supplementary Information, under the corresponding section.]

### Data collection

To minimize the use of ‘black-box’ modeling and extrapolation, extensive data collection was carried out. 167 fishing countries, territories and dependencies (e.g. Greenland) were considered in this study, referred to hereafter as a “country”. For each country, data was collected primarily from governmental sources (reports, censuses, statistical compendium), in addition to datasets already available from international and/or specialized organizations (EU, Tuna RFMOs) and grey literature. All new data collected was checked for consistency and combined with existing FAO-sourced datasets and reports.

New fishing vessel data for 81 countries, representing over 85% of the global marine catch and 95% of the fishing fleet (in number) were collected from the sources provided in Supplementary Table 1^13^, including number of vessels, engine power, length of vessels (LOA), tonnage, fishing gear and activity. Partial time series and punctual data points for a further 78 countries were gathered to complement and cross validate fishing fleet datasets provided by the FAO. The remaining countries used only information from these FAO-sourced datasets, representing less than 5% of the global catch and 1% of the fleet (Table of data sources available at IMAS data repository^13^).

### Separation by sector

As per Rousseau *et al*.^12^, the fleet for each country was separated into three sectors: industrial, artisanal-motorized and artisanal-unmotorized. The definition of artisanal by country was sourced from legal documents, or provided by the data itself^18,19^. It is important to note that the word “artisanal” is not universally used, and this study does not differentiate between artisanal, small scale and subsistence, and applied the most context-appropriate definition, regardless of nomenclature.

When no information about artisanal fisheries were found in national yearbooks and other sources, the definition used by a geographically close country (a neighboring nation using similar fishing techniques background) was used instead. For instance, in Western Africa, pirogues and other canoes were considered artisanal when no other information was present, while in the Maghreb and Arabic Peninsula dhows-type vessels were considered artisanal. In cases where data disaggregated by sectors could not be found and a conceptual separation between artisanal and industrial was deemed unnecessary (i.e. where artisanal vessels differ only from industrial ones by size), the separation focused on motorized and unmotorized segments.

### Time series of number of vessels

While punctual data source and partial time series of the number of vessels could be found for 99% of countries, in most cases, the temporal data records (time series) by country and sector were patchy, and missing records needed to be reconstructed (SI Fig. 1). For each sector and country, the total number of vessels in a country was reconstructed through a Generalized Additive Models (GAM) using the gam package for R^20^. A logistic (sigmoidal) model of the number of vessels by year was chosen to represent the tendency of fishing fleets to follow a carrying-capacity-limited exponential growth^12^.

In countries where environmental, social or climatic events led to the drastic restructure of the fleet (e.g. destruction of the fleet by a cyclone or a civil war), the time series was split into segments, each reconstructed separately. The data for the artisanal fleet of some Small Island Nations was too sparse to allow a GAM-based reconstruction, and was instead reconstructed based on the proxy information of population growth.

### Supplemental analysis for the unmotorized fleet

While data was sufficient to reconstruct the artisanal-unmotorized segment for most countries (76%) using GAM, only punctual information could be found for the remaining 37 countries.

A weakly linear relation (SI Fig. 4) was observed^12^ between the year at which the unmotorized fishing fleet of a country is at its maximum (Y_max_) and the Gross Domestic Product (GDP) per capita of the country at that year. The number of unmotorized vessels was reconstructed in proportion to population growth before Y_max_, and proportional to the rate of change of the motorized artisanal fleet segment after Y_max_.

### Time series of vessel length

In line with FAO classification of vessels by length class (LOA-based), the number of vessels for each country and sector was separated into five different classes of vessel length (under 6, 12, 24, or 50 m, and above 50 m), based on available data.

The time series of ratios for each length class was extrapolated using the same GAM method as for the number of vessels. As per Rousseau *et al*.^12^, the length classes of data-poor countries were estimated as an average of ‘similar countries’, i.e. countries from a similar region and/or level of economic development where the fishing fleet is assumed to have similar characteristics.

### Supplemental capacity information

For each country, sector and length category, time series of the ratio of gear types, following the International Standard Statistical Classification of Fishing Gear (ISSCFG)^17^, were derived from data and interpolated/extrapolated using GAMs. The (gross) tonnage and engine power of each length and gear category was associated with available data, using similar GAM-based time series. The average engine power of each category was allowed to vary with time and region, as described in Rousseau *et al*.^12^, unlike gross tonnage, which is solely based on the measurements of the vessel (length, width, and height) and therefore relatively stable by country, length category, and time period.

### Motor equivalence

The fishing effort used in this study is expressed in terms of engine power (as well as days/hours fishing), therefore for the unmotorized fleet we had to define an ‘engine power equivalent’, i.e. a measure of equivalence between oar and sail and the wattage of a motor.

We assumed that, during the early stage of motorization of a country, the catch per unit of effort (CPUE, here expressed as catch per kW for simplicity) of the total artisanal fleet (motorized and unmotorized) is stable:

1 $$Catc{h}_{Art,y}\propto N{V}_{Art,Unmotor.,y}\times PP{V}_{eq}+{P}_{Art,Motor.,y}$$

with:

- Catch_Art,y_ the total artisanal catch (motorized and unmotorized, including reported landings, discards, and IUU) per year^16^,
- NV_Art, Unmotor., y_ the number of unmotorized vessels per year,
- PPV_eq_ the average engine power equivalent per vessel of the artisanal unmotorized fleet, as compared to the artisanal motorized,
- P_Art, Motor., y_ the total engine power of the Artisanal motorized fleet, per year,

Solving (1) for each country gave a value of engine power-equivalence to the unmotorized fleet. During the early motorization stages of a fishing fleet, the motorized vessels benefit from a ‘novelty effect’ and are often overused compared to their average activity, leading to outliers in activity. Conversely in the later years, the unmotorized vessels are underused, being vastly obsolete. To consider such pattern, we only selected the years when the unmotorized fleet represented 20–80% of the total number of artisanal vessels to calculate the engine-power equivalence of the unmotorized fleet.

### Nominal and effective effort

The number of days at seas was associated to the fishing capacity by sector and gear using datasets created by Anticamara *et al*. (2011) complemented with additional sources (Available at IMAS data repository^13^).

The nominal fishing effort (per fleet segment) was defined as:

2 $$Ef{f}_{Nom}=P\times DAS\times {R}_{Act}$$

with

- Eff_Nom_ the nominal effort (in kW*Days),
- P the total engine power (fleet),
- DAS the number of days at sea,
- R_act_ the ratio of vessels in activity to the total capacity.

Whenever no days at sea data were found for specific fleet segments (country, sector, year, gear), the number of days at sea in the most similar segment was used instead. Data on the activity of the fleet was found only for a quarter of the world’s countries, mainly European countries and dependencies. A mean activity rate of 72% was calculated from this available data, and given to countries without information.

Technological creep, or change in catchability over time^21^, was calculated by determining the biomass-independent increase in CPUE for countries, year, sector and gear. It was determined that the creep was on average 3.5%, although variable in time, region, sector and gear. Creep for the industrial sector was calculated at a mean 1.4% per year, comparable with Palomares & Pauly^21^, while the creep for the artisanal sector was substantially higher at 5%, in line with existing models^22^. Effective effort was defined as the nominal effort multiplied by the creep, using 1949 as a baseline (effective effort in 1949 = nominal effort in 1949, or creep of 0%).

### Mapping

**Fig. 2:**
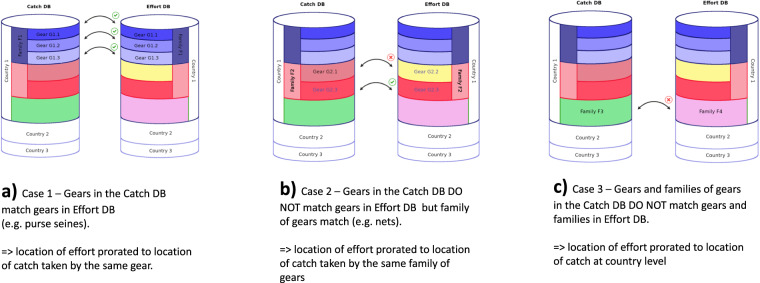
Association of catch location to gear database, using gear as common denominator.

It was hypothesized that fishing effort focuses in and around known locations of the (targeted stocks. As previously mapped catch^16^ data explicitly included habitat (through consideration of e.g. depth, distance to coast, …) and known location of stocks, it was used as a start for locating the effort. Catch was associated with fishing effort by country, year, sector, and gear type (Fig. 2a). Whenever gears used in the effort database were not compatible with those used in the catch data, the overarching general type or ‘family’ of gears (e.g. nets comprising gillnets, liftnets and seines) were used instead (Fig. 2b). In rare cases where effort and catch databases did not agre on the types of gears used, country-level association was used (Fig. 2c).

**Fig. 3:**
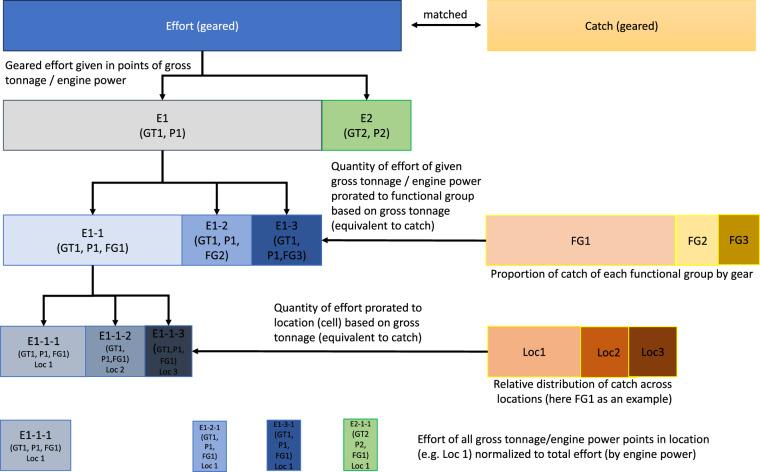
Prorating to functional group, catch and location of catch used in mapping the geared effort.

Once the effort data (i.e. effort of a specific gear, by gross tonnage, length and engine power category, for a year and country) was matched to catch data (i.e. catch of a functional group by a specific gear, as per Watson 2017^16^), the effort was allocated the corresponding functional group of the catch, and matched its location (30 minute cell). Because the catch data was not further broken down to vessel characteristics (engine power, tonnage) within each gear category, the effort needed first to be proportionally allocated to functional groups, by prorating the proportion of catch of each functional group to the gross tonnage of vessels, assuming vessels fish proportionally to their capacity (Fig. 3). The effort in each cell is further standardized across categories of engine power. The relative catch (for each functional group, gear, and vessel category) across grid cells was then used to prorate the spatial distribution of effort. The effort in each cell was further standardized across categories of engine power to ensure that the spatial effort summed to the total amount of effort in each year and category.

The location of the effort was then further refined to consider gear and sector specificities (e.g. bottom trawling banned in some regions and/or impossible at certain depth, unmotorized fleet limited to coastal waters, …). The effort was finally normally spread in surrounding cells, to avoid highly specific hotspots of fishing and mimic the fishermen targeting behaviour.

As fishing hours may be more useful than days at sea to determine the impact of fishing effort on ecosystems, AIS data^8^ was processed to establish conversion factors between days at sea and fishing hours.

Further details of the methodology can be found in the Supplementary Information.

## Data Records

Data records include time series of fishing capacity and effort, by country, mapped effort and conversion factors, and can be accessed at 10.25959/MNGY-0Q43^13^.

The overarching data files are contained in 2 folders, “effort_mapped” and “effort_mapped_country” with 13 descriptive fields (Table 1), describing the nominal and effective fishing effort (in DAS × kW), active and total number of vessels, gross tonnage (GT), and engine power (kW) by year, fishing country, size of vessel, gear, functional group targeted, by grid cells of 0.5 deg of longitude and latitude (centre of the cell):

- “effort_mapped” contains 204 files of mapped fishing effort given by year (1950–2017) and fishing sector (artisanal motorised, artisanal unmotorised, industrial). Preferential use for punctual (one year) view of global fishing effort. The year is not included in the file to reduce size.
- “effort_mapped_country” contains 167 files of mapped fishing effort given by country. Preferential use for time series of individual country.

**Table 1:** Data field descriptors.

Name of field	Description
Year	The year of the fishing effort event
SAUP	Fishing Country Code. Conversion file attached (“SAUPtoCountry.csv”)
NV	Number of active vessels
P	Engine power of the active vessels (kW)
GT	Gross tonnage of the active vessels (gross tonnes)
NomActive	Nominal fishing effort, in kW × days at sea. Only active vessels considered
EffActive	Effective fishing effort, in kW × days at sea. It assumes a variable technological creep (mean 3.5%), with 1949 as comparison value.
Length_Category	Length of the fishing vessels (less than 6, 6–12 m, 12–24 m, 24–50 m, over 50 m)
Gear	Gear used in fishing
Lat	Latitude of the centre of 0.5 degree cell in which fishing takes place
Lon	Longitude of the centre of 0.5 degree cell in which fishing takes place
FGroup	Functional Ggroup of species targetted
Sector	Fishing Sector. Can be APW (Artisanal powered), UP (artisanal unpowered), or I (industrial).

Smaller files are provided for gridded and total effort aggregated by different variables, and conversion files with a usage note are present.

## Technical Validation

### Total number of vessels

**Fig. 4:**
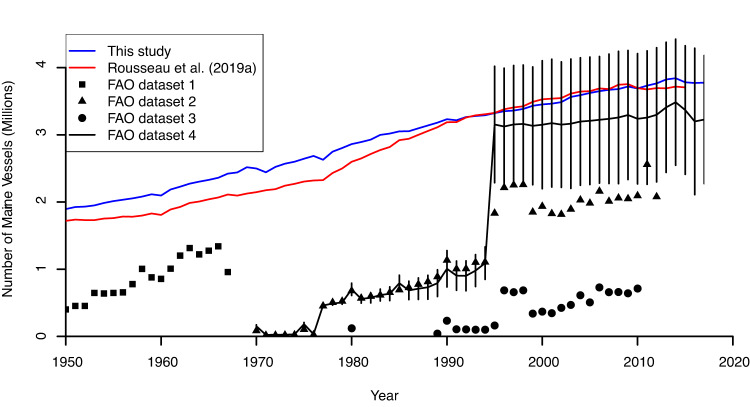
Comparison of the aggregated number of fishing vessels^13^ with previous studies and datasets. Compared datasets include Rousseau *et al*.^12^ and FAO datasets 1–4^23–26^. Vertical bars for FAO dataset 4 account for the uncertainty due to the aggregation of marine and inland fleets.

The dataset^13^ was compared with, amongst others, FAO^23–26^ and AIS data^8^, to ensure completeness, assess robustness, and to facilitate comparability. At the highest level of aggregation, the number of vessels show similar values as previous reconstruction studies (Fig. 4). Considering that the most recent FAO dataset^26^ is not fully disaggregated between marine and inland fleets, leading to high uncertainty in the number of specifically marine vessels in Southeast Asia and along the Nile, the number of vessels in this study is within the uncertainty margins of FAO data. Earlier higher values in this study are the consequence of more complete data sources, the FAO data having historically focused on industrial fleets and/or fleets of industrialised countries^12,14,27,28^. A higher number of vessels in this study compared to previous ones can also be explained by a more in-depth analysis of the European fishing fleet and less reliance on the EU registrar for earlier years.

**Fig. 5:**
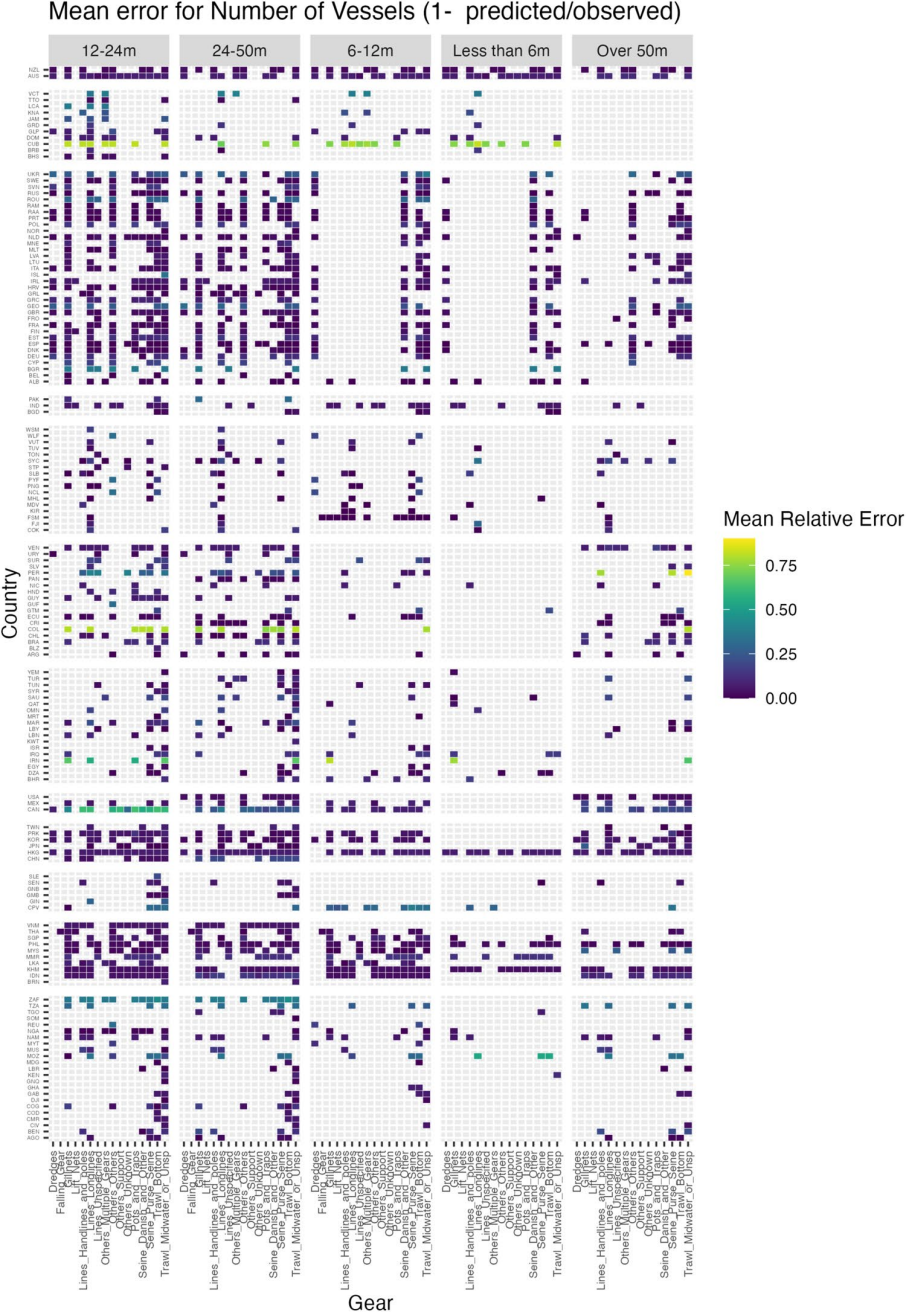
Mean relative error by gear, country and vessel size in reconstructed data versus raw data for number of vessels (industrial sector). Dark blue indicates low error (close to zero); green/yellow indicates higher errors. Definitions of International Standards Organization alpha-3 country codes can be accessed here: https://www.iso.org/obp/ui/#search.

Overall, the effectiveness of the reconstruction using generalised additive models (GAM) for each combination of country, sector, fleet, and vessel size through time was good, with low mean relative errors (less than 5%) for most combinations (Fig. 5). Higher errors demonstrate regions in which data coverage could be improved and needs further research.

### Comparison to AIS data

**Fig. 6:**
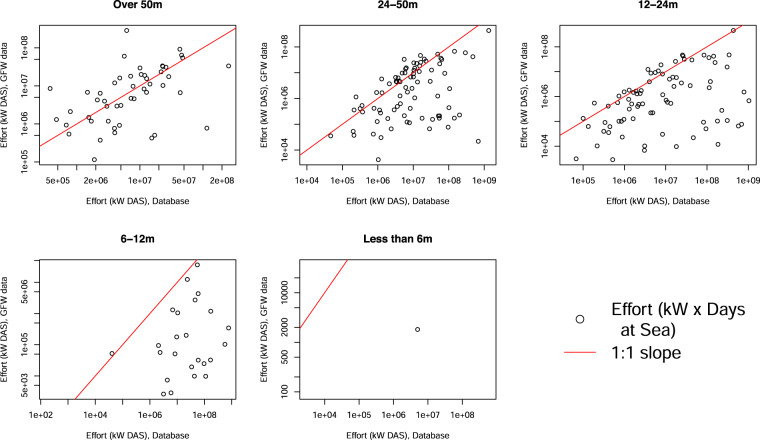
Comparison of effort (in kW × day at sea) between this study^13^ and AIS-derived data^8^, by size of the vessel, for 2017. Each dot corresponds to one country, the red line indicates where a theoretical perfect agreement (1:1 ratio) between the datasets would be. AIS data downloaded from Global Fishing Watch^8^.

The allocation of length categories, engine power and days at sea can be compared to AIS datasets^8^. Nominal effort of the largest vessels is comparable with AIS-derived data, while the lack of small-vessel data is highlighted (Fig. 6).

**Fig. 7:**
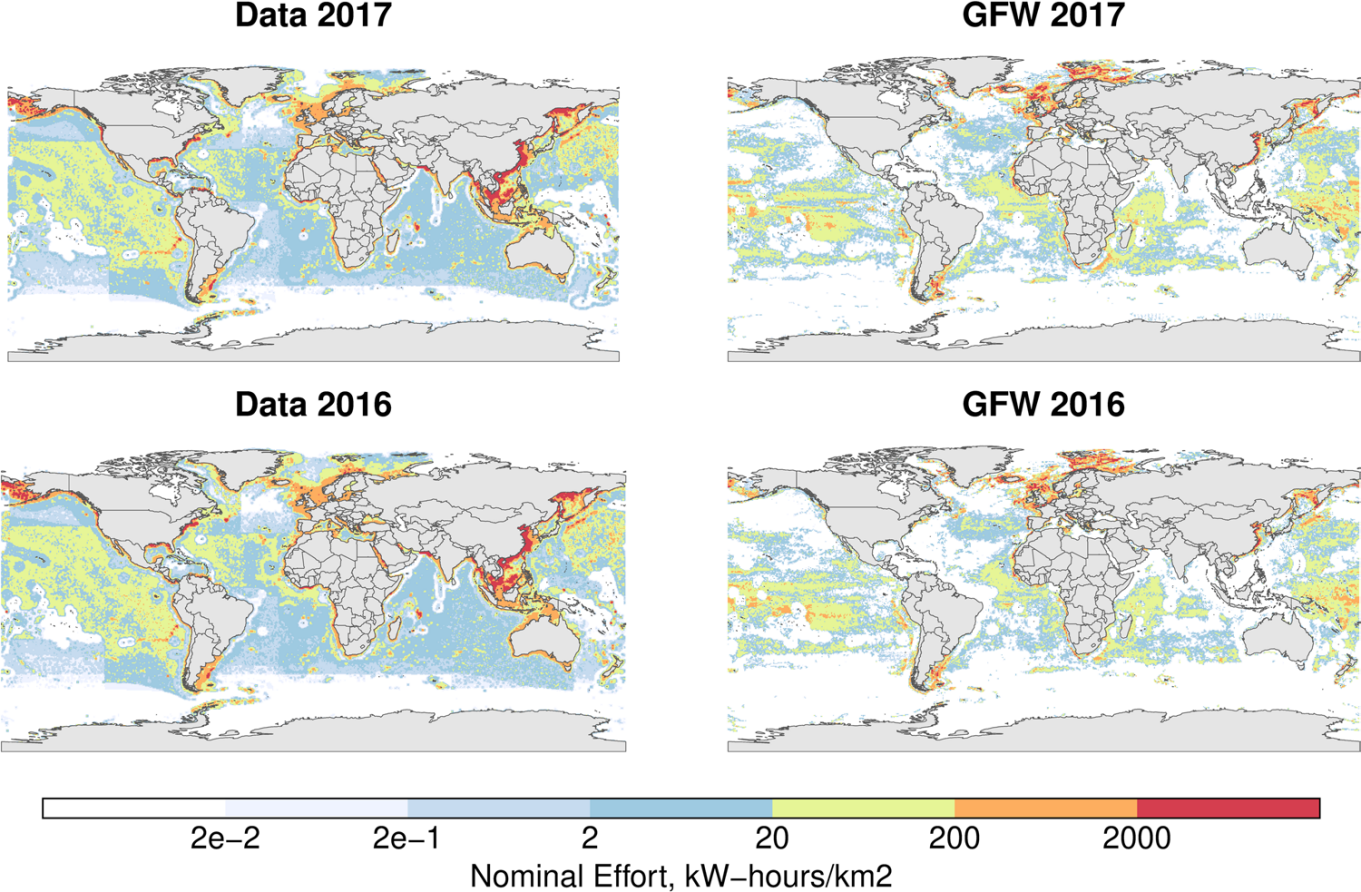
Comparison of the mapped nominal effort (in kW.hr/km2) between the study and AIS-derived data^8^. AIS data downloaded from Global Fishing Watch.

Finally, the gridded effort^13^ can be compared to maps generated by AIS data^8^, as a validation of both the catch-based mapping of the effort and previous mapping of catch according to physical and environmental parameters^16^. While the effort data is more widespread than the AIS-derived data, most hotspots of fishing are highlighted in the statistical comparison of the mapped effort (Fig. 7). Both datasets are shown to be highly correlated and directly comparable (i.e. cell by cell) to the order of 30% (Table 2). Direct comparison of maps, however, does not account for distance between regionalization, and these results underestimate the similarity between the datasets at larger spatial scales, as well as remain influenced by the statistical methodologies used for comparison, accounting for the differences between Spearman correlation and V-measure.

**Table 2:** Comparison of mapped AIS and effort datasets^13^ for 2016 and 2017, using direct correlation and V-measure^33^.

Year	Length_Category	Spearman	p_value	Vmeasure	Homogeneity	Completeness	GoF
2016	6–12 m	0.255	6.34E-17	0.790	0.671	0.960	0.443
2017	6–12 m	0.260	2.01E-18	0.789	0.672	0.956	0.444
2016	12–24 m	0.350	0	0.369	0.330	0.419	0.423
2017	12–24 m	0.355	0	0.368	0.336	0.406	0.427
2016	24–50 m	0.225	0	0.294	0.291	0.298	0.328
2017	24–50 m	0.195	0	0.297	0.293	0.301	0.322
2016	Over 50 m	0.227	0	0.267	0.318	0.231	0.073
2017	Over 50 m	0.224	0	0.268	0.317	0.232	0.068

Results for the category 6–12 m are unreliable and inflated due to few data points in the AIS datasets for that category.

**Fig. 8:**
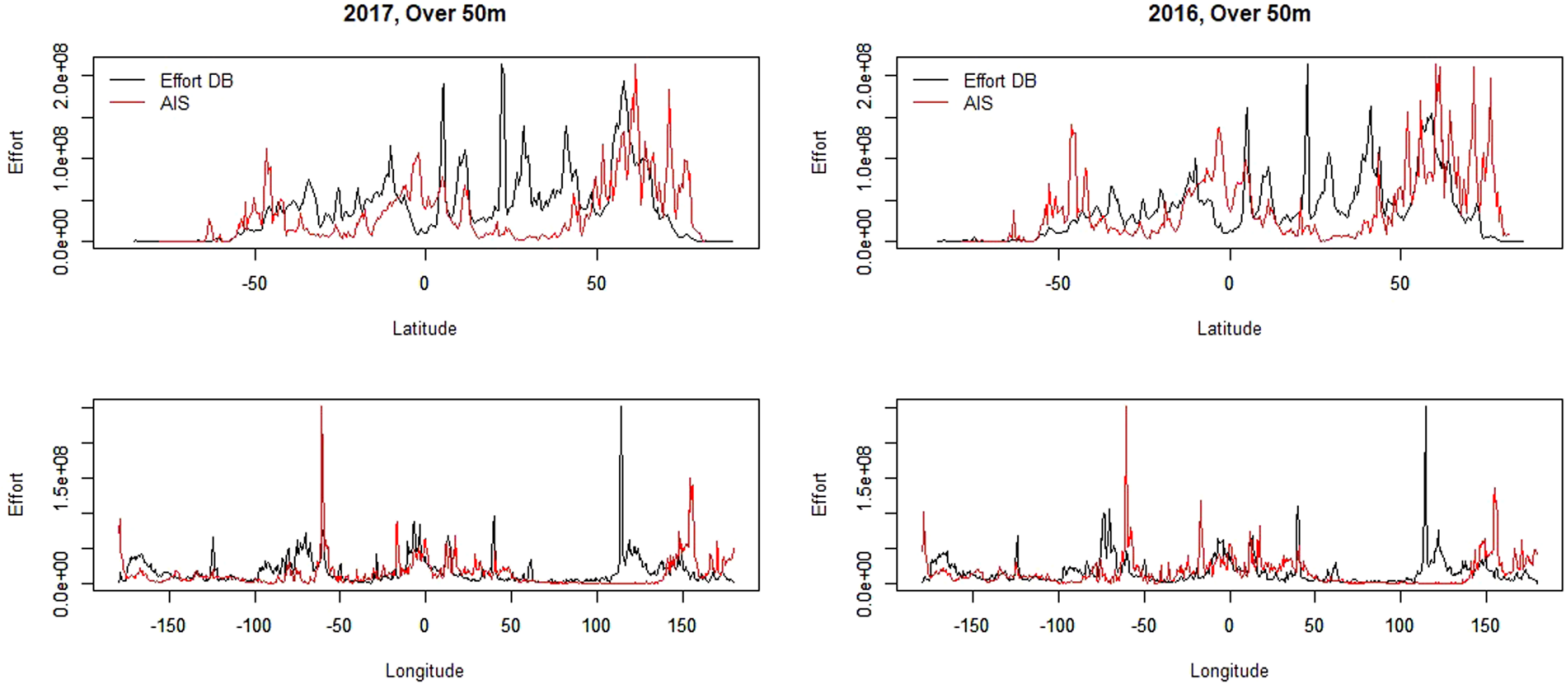
Comparison of the mapped nominal effort (in kW.hr) between the study^13^ and AIS-derived data^8^, aggregated by longitude and latitude. AIS data downloaded from Global Fishing Watch.

Aggregated data by longitude/latitude separately, while lower in resolution, allows for better comparability of the datasets (Table 3, Fig. 8). It shows in particular the increased correlation between datasets with increased length of vessels, representing the higher level of spatial knowledge associated with larger vessels (often more scrutinized and more covered in AIS datasets).

**Table 3:** Cosine values comparing AIS^8^ and effort datasets^13^, by year, length category, longitude, and latitude.

Lat	Lon	Year	Length_Category
0.105	0.192	2016	6–12 m
0.129	0.195	2017	6–12 m
0.335	0.407	2016	12–24 m
0.357	0.420	2017	12–24 m
0.654	0.589	2016	24–50 m
0.684	0.598	2017	24–50 m
0.645	0.477	2016	Over 50 m
0.677	0.499	2017	Over 50 m

## Usage Notes

**Fig. 9:**
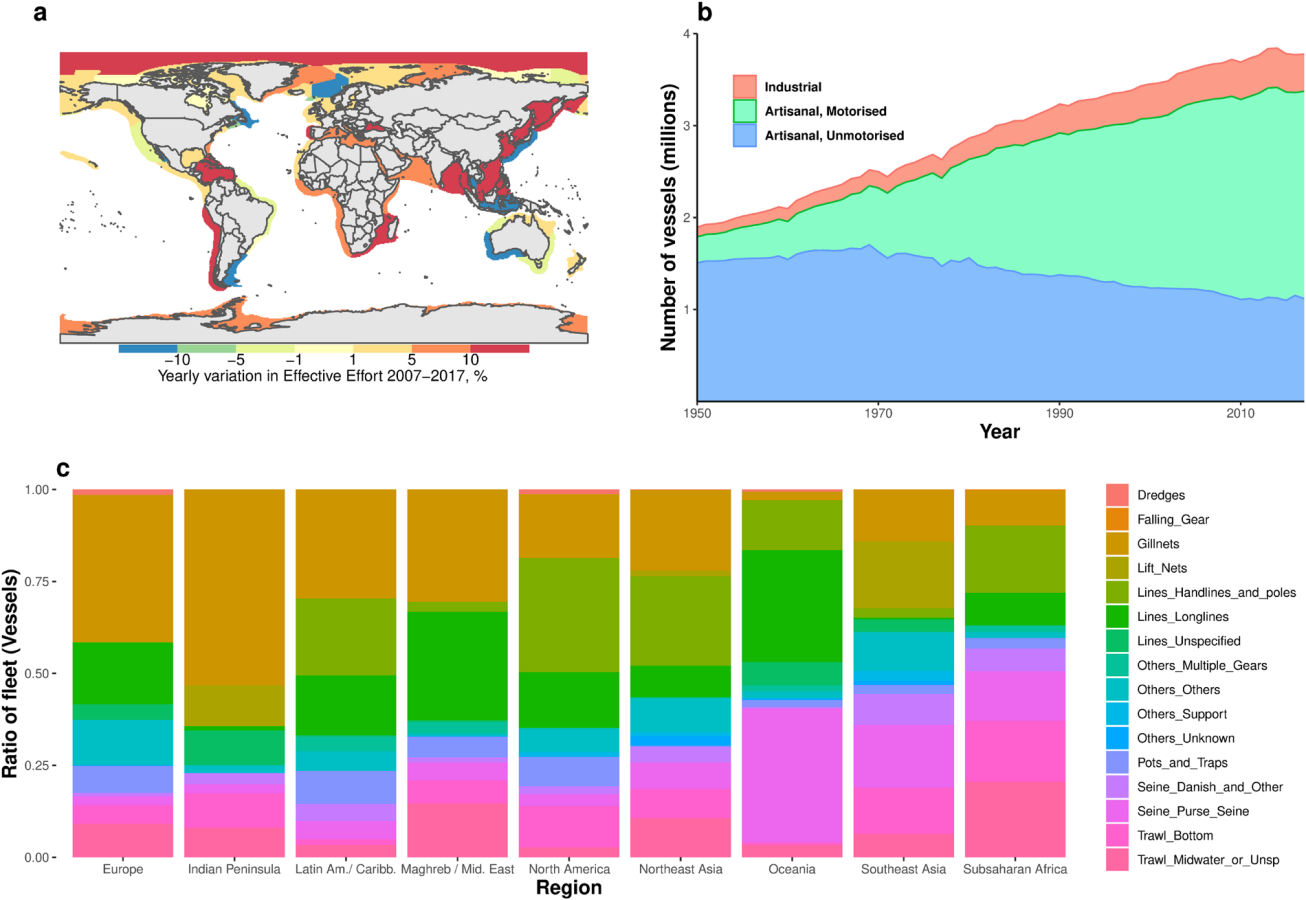
Extract of data contents. (**a**) Map of yearly variation in effective fishing effort 2007–2017 in the world Large Marine Ecosystems. (**b**) Number of vessels by fishing sector, 1950–2017. (**c**) Ratio of fishing vessels gears employing specific gears by region, 2017.

Earlier published and unpublished versions of the file have provided fleet capacity and effort to a range of researchers, used as fishing mortality^29^, for ecological output and modelling^30,31^, and validation of economic and social modelling of fishing activities^32^. A few other examples of applications of the data are demonstrated below. Figure 9a shows the average yearly variation (2007–2017) in effective fishing effort, by Large Marine Ecosystem (LME). Mapped effort allows to input fishing mortality in mapped ecosystem models, whether global, regional, large marine ecosystem or country level. Figure 9b shows the size of artisanal and industrial sectors in the world fleet, their evolution in time and the degree of motorization. Country and region-aggregated data *has been used to reconstruct time series employment and* Catch per Unit of Effort. Figure 9c shows the relative usage of various gears in different regions, indicator of the level and type of technology used in fisheries.

The data in its current form contains aggregated yearly of fishing effort in fishing hours and days at sea and disaggregated files of effort in days at sea only. Users interested only in the activity of fishing itself in its full gridded version could transform the effort in Day at sea × kW to days at sea only, or convert to fishing hours using provided the conversion file.

The data is published under a Creative Common Licence, and can be used by anyone with proper citation.

### Supplementary information

Supplementary information

## Data Availability

Data analysis was carried using readily available (open source) R code and packages. Code used in data extrapolation and interpolation is provided in the Supplementary Information. Example code for effort mapping and to explore fishing capacity (R-shiny) is provided in https://github.com/Global-Fishing-Effort/RousseauEtAl2023.
