# Supplementary material for: A database of mapped global fishing activity 1950–2017
Source: Sci Data. 2024 Jan 8;11:48. doi: 10.1038/s41597-023-02824-6 (PMC10774419; doi:10.1038/s41597-023-02824-6)
Supplement: Supplementary file 1 — Supplementary information [file 41597_2023_2824_MOESM1_ESM.pdf]

Supplementary Information for Rousseau et al. '*A database of mapped global fishing activity, 1950-2017*'

Table of Contents

Data collection 2

Separation by sector 3

Time series of number of vessels 4

Supplemental analysis for the unmotorized fleet 5

Time series of vessel length 8

Gearing, gross tonnage and engine power 9

Engine power equivalent 10

Days at sea and nominal effort 13

Association Catch – Nominal effort 14

Mapping Effort 14

Converting Days at sea to Fishing hours 14

Technological creep and effective fishing effort. 17

Code Availability 19

Data Citations 20

References: 20

Data collection

The global capacity database is comprised of a combination of FAO data, external data collected and reconstructed or "estimated" values. For each of 167 countries and territories considered in this study, the data was compiled in three steps:

- Collection of data from government sources, including yearbooks, census, statistics websites/compendium and various ministries (fisheries, agriculture, economy, …).
- Collection of data from international datasets (e.g. EU fleet register, Tuna commissions vessel lists, …) and scientific/grey literature.
- Cross validation of data between sources and with FAO datasets to remove outliers and ensure proper classification of data.

Various data sources were used to determine time series of number of vessels by sector (Fig. S1, processes 2 to 4), separation of capacity by length category (Fig. S1, process 5) and association of gear, tonnage, and engine power (Fig. S1, process 6). The number of vessels (by sector) came primarily from national registries, census, yearbooks, and governmental reports/websites, along with selected published and grey literature (See data repository).

The vessel data for 81 countries, representing over 85% of the global marine catch and 95% of the fishing fleet (in number) were collected from these sources (Figure S1). Partial time series and punctual data points for a further 78 countries were gathered to complement and cross validate fishing fleet datasets provided by the FAO. The remaining countries used only information from these FAO-sourced datasets, representing less than 5% of the global catch and 1% of the fleet (See data repository).

Average vessel characteristics (length, gear, tonnage, engine power) were further sourced from FAO datasets and Regional Fisheries Management Organizations (RFMOs) vessel lists, including registers from tuna RFMOs (IOTC, ICCAT, WCPFC, IATTC, CCSBT[1]), European Union fleet register[2], and others (GFCM[3], NEAFC[4], NPFC[5], SIOFA[6], SPRFMO[7]).

---

[1] http://clav.iotc.org/browser/search/

[2] https://webgate.ec.europa.eu/fleet-europa/index_en. Unlike Rousseau et al. [1] which used the EU register as primary source of information, leading to underestimates of the fleet in earlier years, this study used country specific data to reconstruct the number of vessels, and EU data to associate vessels characteristics to each countries' fleet.

[3] https://gfcmsitestorage.blob.core.windows.net/contents/DB/GoL/Html.htm

[4] https://www.neafc.org/managing_fisheries

[5] https://www.npfc.int/compliance

[6] https://www.apsoi.org/mcs

[7] https://www.sprfmo.org/web/public/vessel

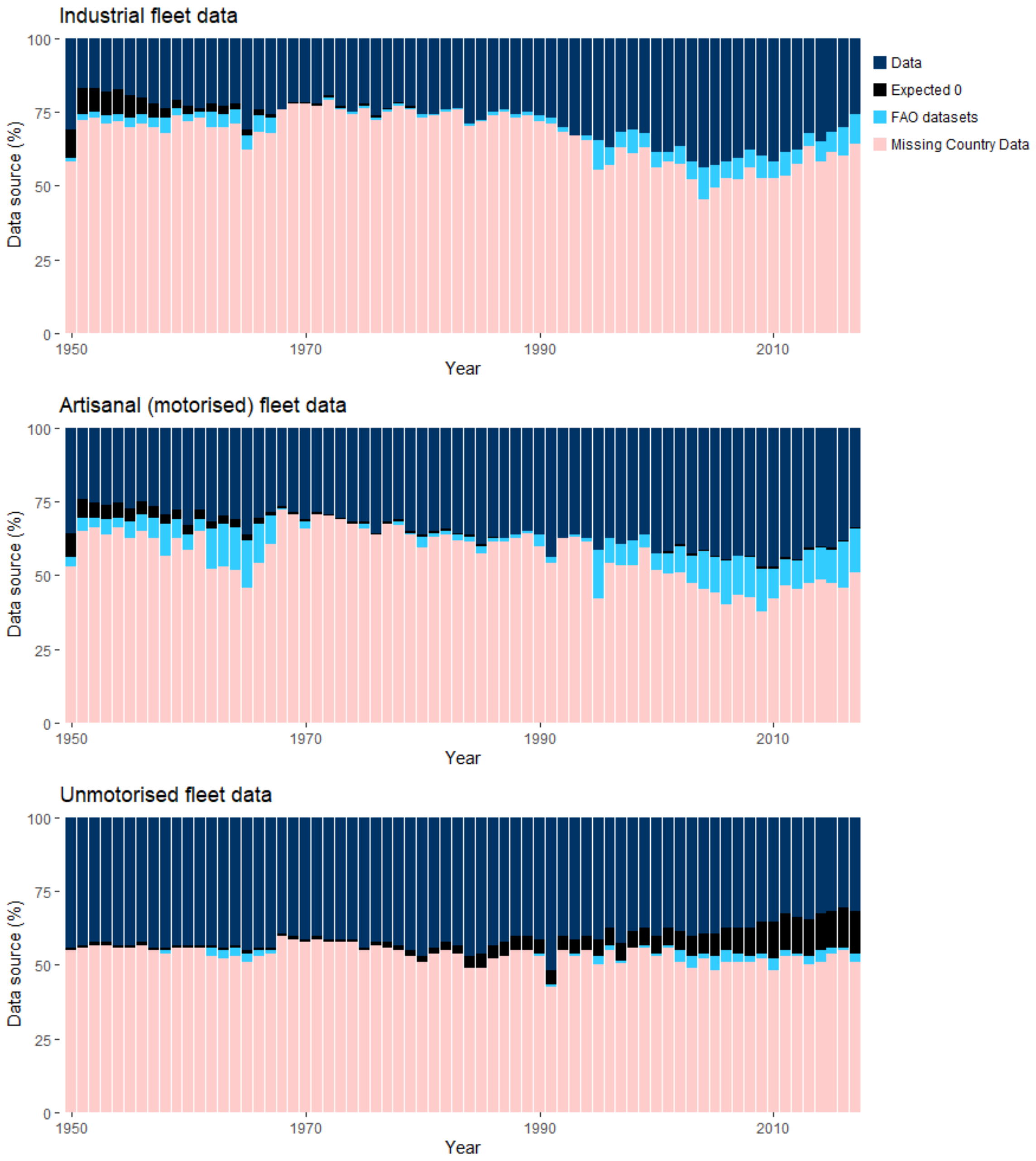

Figure S1. Proportion of data sourced used to reconstruct the number of marine fishing vessels, by sector, for 167 countries and territories. Each data source point (country, year, sector) is weighted equally regardless of the number of vessels it represents. "Data" refers to data points from governmental sources and / or grey literature. "Expected 0" are missing data at years when the number of vessels for that country and sector should be 0 (e.g. number of industrial vessels prior to the introduction of the motorised boats in the country).

Separation by sector

The data source for each country and year was categorized by sector, so that to obtain a (often incomplete) time series of number of vessels by country and sector 1950-2017. The fleet data was systematically separated into 3 independent sectors (when appropriate): artisanal unmotorized

(referred hereafter as “unmotorized” or “unpowered”), artisanal motorized, and industrial. Available vessel characteristics (length, gross tonnage, engine power and/or gear used) were also allocated to these sectors wherever possible.

The definition of artisanal[8] by country was sourced from legal documents, or provided by the data itself [1,2]. When no information about artisanal fisheries were found in national legal documents, the definition of a close country (neighboring nation of similar fishing techniques background[9]) was used instead. In some cases, a sectorial separation was deemed unnecessary as the legal definition did not fit the type of data found, and the separation focused on unmotorized vs. motorized, length or tonnage categories. For instance, European fleets use a cut-off definition of small-scale at 12m which is not representative of the continuum in boat length, nor does it correspond to the format of the historical data collected for individual countries, while the separation between motorized and unmotorized fleets was clearly given in the data.

Data for artisanal fleets (motorized and unmotorized) could not be found for a few countries around the Persian Gulf and the Gulf of Aden, and it was assumed that the marine fleet of these countries was entirely industrial, with artisanal fleets limited to inland/river waters.

Time series of number of vessels

The combined new and FAO datasets for the vessel numbers by country is sector was patchy, with over 50% of time series missing in most cases (Figure S1, detailed in Table in data repository).

Typically, missing data are inferred with linear or logistic/sigmoidal models “anchored” to existing data points [1,3]. Extrapolating beyond the first and last year of available data is typically implemented using ARIMA-based models [1,4]. While the predictive power of ARIMA is good, it is an error-sensitive approach which leads to high uncertainty interval in predicted results.

Here, the total number of vessels in each sector for each country was reconstructed through a regression implemented with Generalized Additive Models (GAM) using the gam package for R [5]. A logistic (sigmoidal) model [10] of the number of vessels by year was chosen to represent the tendency of fishing fleets to follow a carrying-capacity-limited exponential growth [1].

Unlike previous studies, the number of data points and temporal scope of sources was sufficient to avoid ARIMA-based extrapolation, and the number of vessels per country and associated error could be inferred directly from GAM models (Figure S2, left).

---

[8] As noted in Rousseau et al. [2], “artisanal” is not universally used. This study does not differentiate between artisanal, small scale and subsistence, and applied the most appropriate definition, regardless of nomenclature. Recreational fishing fleets were ignored.

[9] For instance, in Western Africa, pirogues and other canoes were considered artisanal when no other information was present, while in the Maghreb and Arabic Peninsula dhows-type vessels were considered artisanal.

[10] Gam parameter using a gaussian family with logistic link.

Prior to the first year of motorization and after the last year on unmotorized vessels, values for the motorized and unmotorized segments respectively were fixed at 0.

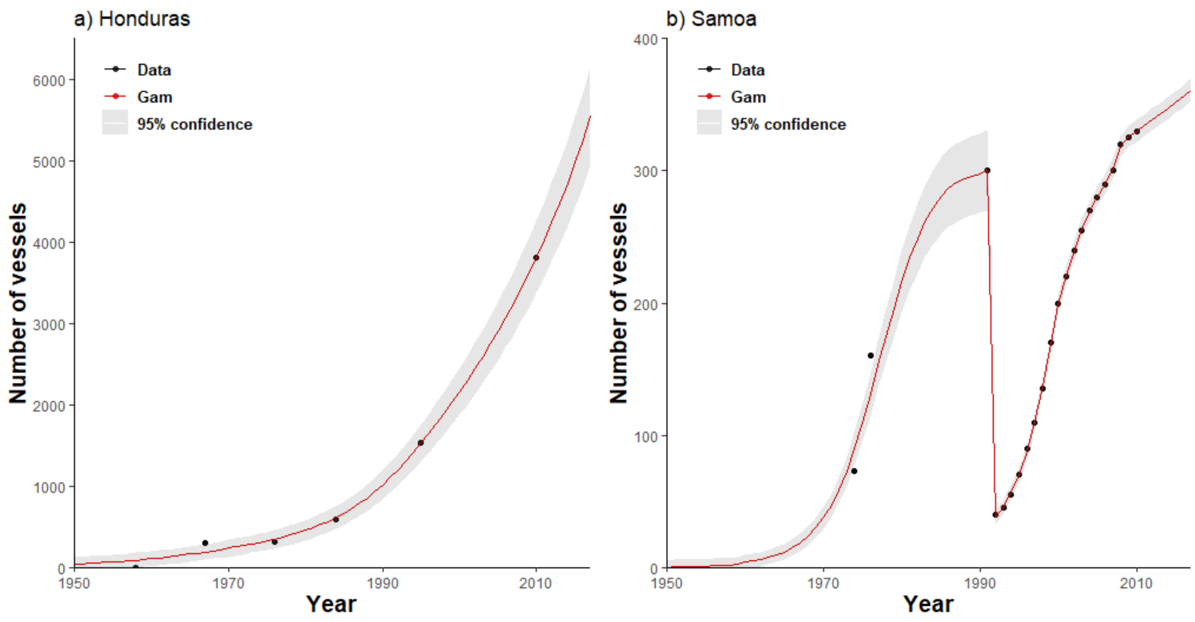

Figure S2. Reconstruction of the number of artisanal (motorised) fishing vessels in Honduras and Samoa, based on available data. The greyed area corresponds to the 95% confidence interval of the GAM model. The fishing fleet of the Samoan Islands was almost completely destroyed by a cyclone in 1991 and the data series split in 2 accordingly.

In countries where specific events led to drastic changes in the fishing fleet (e.g. destruction of the fleet by a cyclone or civil war, nation-wide restructuration of the fleet or ban on specific fisheries), the time series were split into various segments, and reconstructed separately (Figure S2, right). Whenever relevant data points bounding the fits could only be found for years outside the study period (e.g. data for the 1940s prior to the split of Germany), the time series was extended to include relevant data. In a handful of cases (typically the artisanal sector of pacific island nations), only one to two data points were found [6] , and the time series was reconstructed based on population growth.

Supplemental analysis for the unmotorized fleet

The non-motorized (unpowered artisanal) fleet of 119 countries could be reconstructed using the GAM interpolation / extrapolation (or lacking an unmotorized fleet due to motorization fully completed by 1950). The remaining 37 countries, however, did not present enough data points (<4 and/or not spread temporally) to allow the use of this method.

Rousseau et al. (2019a) showed a relationship between the year at which the unmotorized fishing fleet of a country reaches its peak number (Figure S3) and the Gross Domestic Product (GDP) per capita of the country at that year. This relation was expended to the available data of 119 countries and shown to be weakly linear model (Adjusted-$R^2 = 0.421$, $p < 1e^{-4}$, Figure S4).

While it seems counterintuitive that the level of GDP 'required' for a country to translate into motorization increases with time, it can be explained by a delay between economy and technology: the GDP increases faster than technological implementation, leading to an ever increasing 'GDP threshold' for motorization.

This relationship was used to approximate the year at which each country would reach their peak unmotorized fleet. The number of unmotorized vessels was then reconstructed in 2 steps:

- After the peak year, the number of unmotorized vessels was estimated from the rate of change in the number of motorized (artisanal) vessels, with the assumption that the motorized fleet will "replace" the unmotorized fleet. For countries with at least 2 data points after the peak, the rate of change in the number of unmotorized vessels between these points was compared and inferred to the rate of change in the number of motorized artisanal vessels, and the number of unpowered vessels reconstructed accordingly. For countries where only one data point (unmotorized) was available, the rate of change in the number of unmotorized vessels was estimated from similar countries (socioculturally and economically close).
- Prior to the peak, the number of unmotorized vessels was estimated increasing proportionally with population growth.

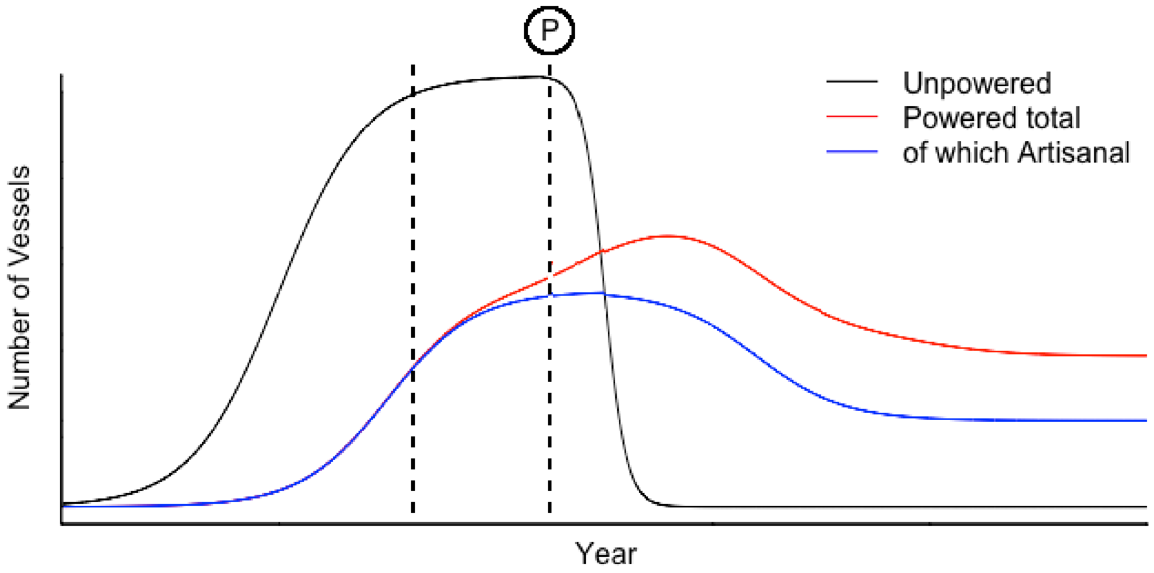

Figure S3. Conceptual representation of the motorization of the fishing fleet of a given country. (P) represents the peak number of vessels of the unmotorized artisanal portion of the fishing fleet.

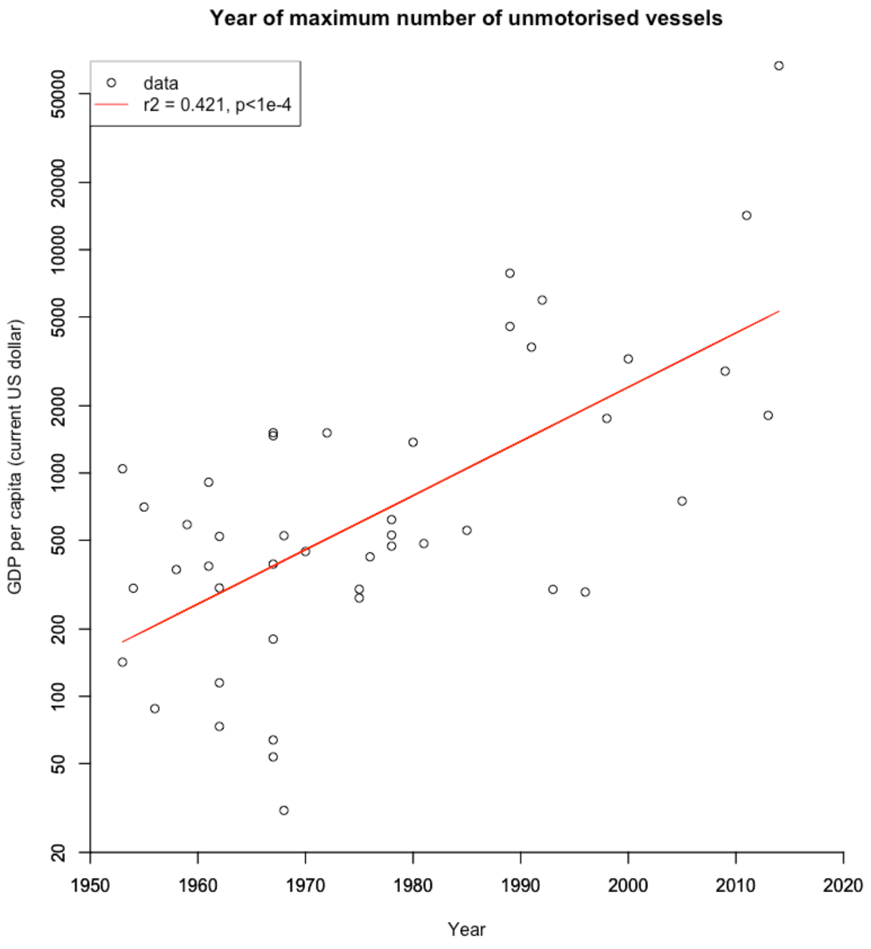

Figure S4. Gross Domestic Product (GDP) per capita at the year of maximum number of unmotorised vessels in the country's marine fisheries. Each point represents one country, the year at which it reached its peak unmotorised fleet and its associated GDP per capita (current US dollar) at that year.

Time series of vessel length

In line with the overall FAO classification of vessels by length (LOA)[11] category, the data (number of vessels) was separated into 5 different categories of vessel length (under 6, 12, 24, or 50m, and above 50m).

For each country and sector, the ratio of vessel in each category was reconstructed through GAM of available data (Figure S5, left), mostly from RFMOs, European Union and FAO datasets. While these datasets often did not fully capture the number of vessels by country and sector, any data point accounting for 50% of the fleet or more at that year were considered representative of the whole

[11] Various measure of the boat length exist: Overall length (LOA), length of hull, length of deck, length between perpendiculars (LBP), ... Each country and RFMO have different requirements for reporting vessel length and their units, with LOA and LBP the most commonly used. While each measure of length gives slightly different values, this study does not differentiate between each and assume length to be LOA.

fleet. As per Rousseau et al., (2019a)[12], the length categories of data poor countries were estimated as an average of 'similar countries', i.e. countries of similar region and/or economic development were the fishing fleet is assumed to have similar characteristics (Figure S5, right).

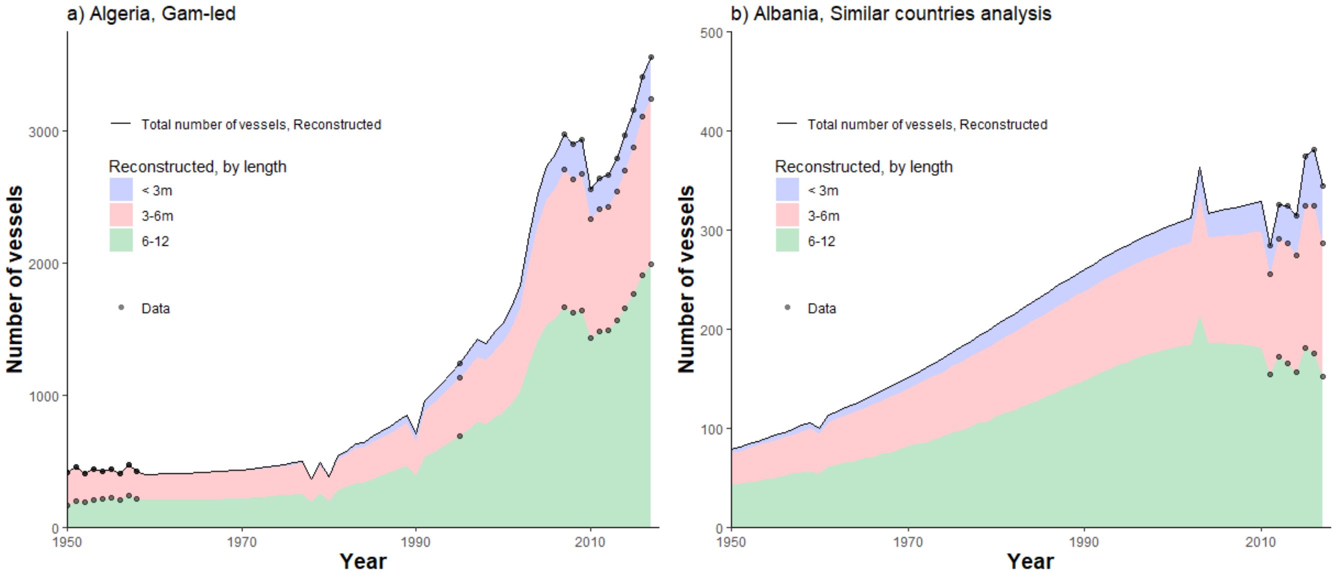

Figure S5. Reconstruction of the number of vessels by length category for the motorised artisanal fleets of Algeria (left) and Albania (right). The total number of vessels was reconstructed following the methods described in 2.2.3. Algeria's separation by length category was then achieved by extrapolating available data with gam models. Albania's separation was achieved by comparing the average proportion of vessels of various length to that of countries presenting similar economic development. The under 3m was combined in final results under the label "under 6m".

Gearing, gross tonnage and engine power

For each country, sector and length category, time series of the ratio of gear types, as following the International Standard Statistical Classification of Fishing Gear (ISSCFG)[13] were created.

[12] Rousseau et al. [1] used this method to categorise the total number of vessels by engine power category.
[13] http://www.fao.org/cwp-on-fishery-statistics/handbook/capture-fisheries-statistics/fishing-gear-classification/en/
Due to the vast majority of countries using generic families of gears (e.g. 'trawls') instead of specific gears types (e.g. 'beam trawls' vs 'otter trawls'), only the gear family, or upper level of ISSCFG, were used.

These time series were completed using GAM and similar countries analysis, as with the length (Section 0, Figure S5), leading to a dataset of the number of vessels by country, sector, year, length category and gear. Gear categories for unmotorized fleets were often lacking, and it was assumed that the segment was akin to the motorized artisanal of the country, whenever data could not be found.

This assumption presents some limitations in the accuracy of gear distribution of the unmotorized fleet. In fact, 2 broad cases can be established:

- The motorized artisanal fleet is ‘retrofitted’ with engines. This occurs in countries and regions where motorization of the fleet did not start till the 1950s or after. In these regions, the marine fishing fleet and fishing grounds were already established prior to motorization. These are fleets such as pirogues, sampans and others, and the introduction of engines mattered mostly for the efficiency of the fleet rather than its aims. Most artisanal fleets of Asia and Africa (excluding Maghreb and Middle East) belong to this category.

- The motorized fleet is a totally different design than the unmotorized fleet. This occurs in regions where the motorization of the fleet is older, or the style of boat in not compatible with engine retrofitting. The motorization led to the development of distant water fleets and vessels of different shape and aims.

In the former case, the assumption of a unmotorized fleet similar to that of the motorized artisanal is coherent and would not increase drastically the uncertainty from associating gears with fleet sector. In the latter case, however, it is possible that the unmotorized fleet does/did not in fact target similar species than the motorized artisanal segment, leading to a misconstrued gear distribution. Since the present study is limited to 1950 and onwards, the vast majority of the unmotorized fleet of the world belong the first case, and uncertainty in the gear distribution of the unmotorized fleet should not be of consequence. This would not be the case, however, if the temporal scope would extend to the early 20th century.

The (gross) tonnage and engine power of each length and gear category was associated with available data, using similar GAM-extrapolated time series. Unlike gross tonnage, which is solely based on the measurements of the vessel (length, width, and height) and therefore relatively stable by country, length category, and time period, the average engine power of each category was allowed to vary with time and region, as described in Rousseau et al. (2019a).

Engine power equivalent

The fishing effort used in this study is expressed in terms of engine power, and for the unmotorized fleet it means defining an ‘engine power equivalent’, i.e. a measure of equivalence between oar and sail and the wattage of a motor. Rousseau et al. (2019a) assumed that the unmotorized fleet was

exactly half as efficient as the smallest components in the motorized artisanal fleet of a country[14]. While this approximation was practical, it led to vast uncertainty and probable underestimates on the effort of the unmotorized fleet. In particular, very little distinction was made between the different unmotorized vessels of different countries/regions: some countries' unmotorized fleet rival with the motorized one (such as the rafts in Taiwan or the pirogues in Sub Saharan Africa), questioning the "half as efficient" assumption.

This study takes a different approach to compare the engine power equivalent of the unmotorized fishing fleet across countries: it was assumed that, during the early stage of motorization of a country, the CPUE (expressed as catch per kW for simplicity) of the total artisanal fleet (motorized and unmotorized) would be stable:

$$Catch_{Art,y} \propto NV_{Art,Unmotor.,y} \times PPV_{eq} + P_{Art,Motor.,y} \quad (1)$$, with:

- $Catch_{Art,y}$ the total artisanal catch, per year,
- $NV_{Art,\ Unmotor.,\ y}$ the number of unmotorized vessels per year,
- PPV_{eq} the engine power equivalent of the artisanal unmotorized fleet, as compared to the artisanal motorized,
- $P_{Art,\ Motor.,\ y}$ the total engine power of the Artisanal motorized fleet, per year,

The catch of the total artisanal fleet (motorized and unmotorized), including reported, discards and IUUs [7,15] was associated to the number of vessel by country and year. Only the years were the unmotorized fleet represented 20-80% of the total number of artisanal vessels were kept, to ensure comparability of the motorized and unmotorized segments. For each country, a linear model of the catch depending on the number of vessels was reconstructed (Formula 1).

This allowed to reconstruct the engine power equivalent of the unmotorized fleet per country (Figure S6). For Georgia (lack of artisanal catch data), Madeira, and the Azores (Catch data aggregated with Portugal), the mean value of 5.15kW was allocated to their unmotorized fleet. Although high variability was found by country, this mean engine power equivalent is similar to the one used in previous studies [1].

[14] That is, if a country used 4kW outboard vessels, the unmotorised vessels were given a power equivalence of 2kW per vessel.

[15] In recent years, there has been a push in the fisheries science community to use the catch data from the Sea Around Us (http://www.seaaroundus.org/) over any other dataset, including that of the FAO. As the methods used by the Sea Around Us are ultimately based on FAO data and previous reconstruction work, there is in fact little difference in the total catch by country between the Sea Around Us and Watson's datasets. The choice of the latter as source of catch for this study stems from 1) a higher transparency in data sources and lower reliance on "private conversations", 2) a more consistent approach to extrapolation of FAO catch data, 3) a more similar definition of artisanal used compared to this study and a higher compatibility of the catch and capacity datasets by country, and 4) data spanning 1950-2019, removing the need for extrapolating catch for latter years.

Combining with the number of unmotorized vessels for each country, the 'power' of the global unmotorized fishing fleet has added a yearly average of 6 million kW of capacity to the capacity of the industrial and artisanal motorized fleets (Figure S7, left). In relative proportion, this indicates that in 1950, 18% of the fishing capacity (expressed in total engine power) was a result of the unmotorized fleet (Figure S7, right), decreasing continuously to represent less than 5% in 2017.

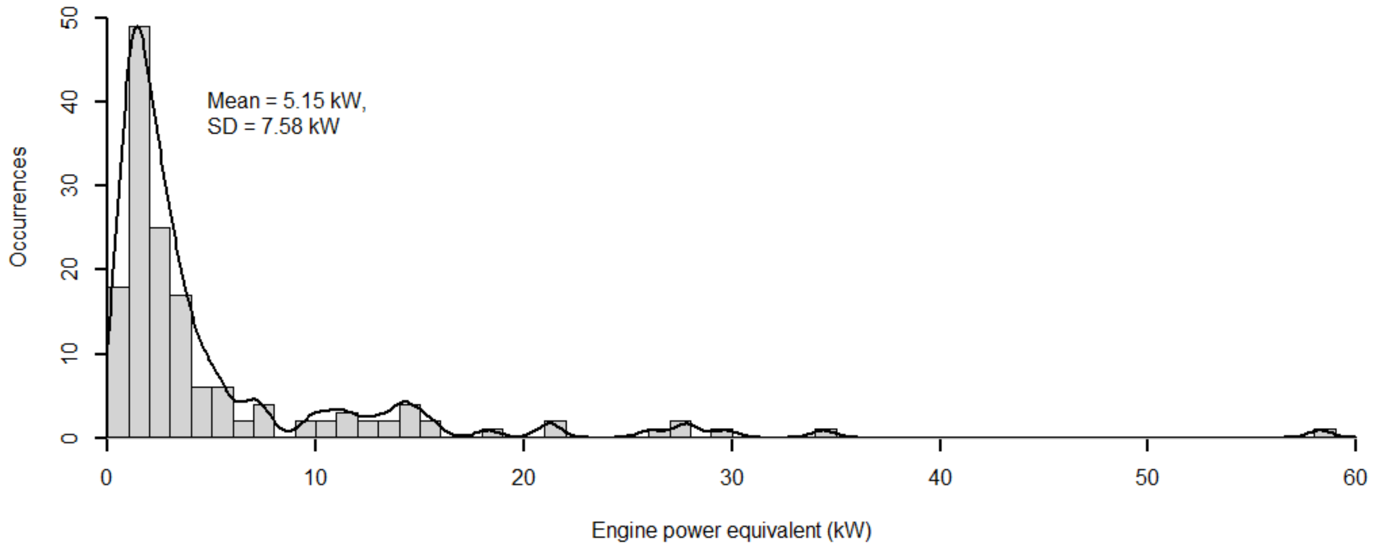

Figure S6. Calculated engine power equivalent of the unmotorised fleet. Each occurrence corresponds to a country.

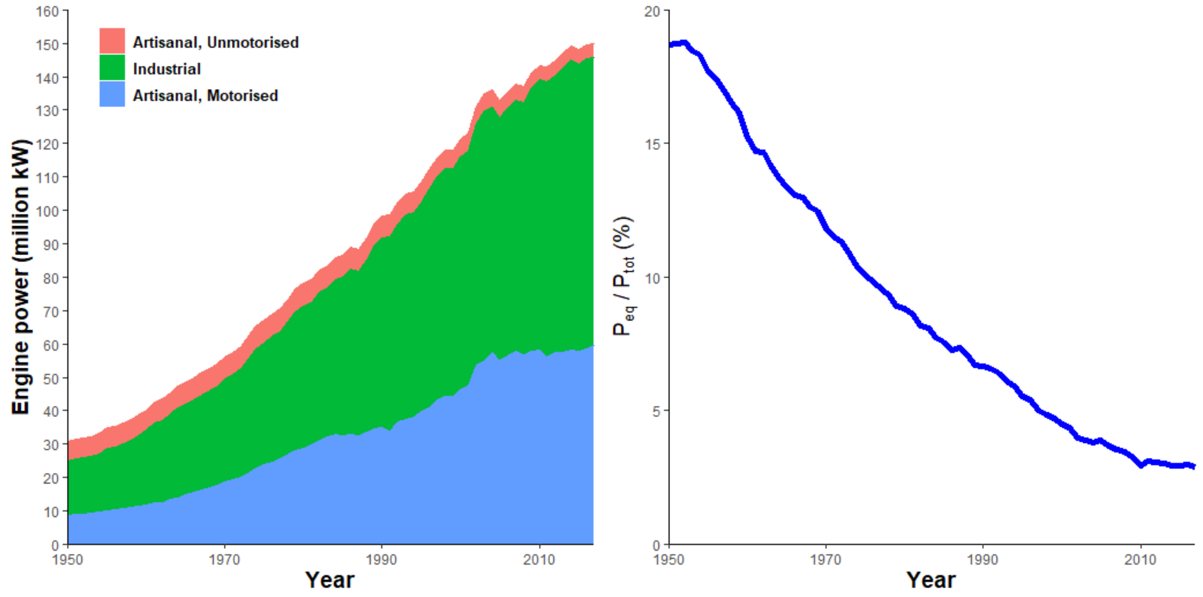

Figure S7. Total engine power and engine power equivalent of the global marine fishing fleet (left) and ratio of the engine power equivalent of the unmotorised fleet to the total engine power (right).

Days at sea and nominal effort

The number of days at seas was associated to the fishing capacity by sector and gear, using datasets created by Anticamara et al. (2011) completed with punctual sources (see data repository). Whenever no days at sea data were found for specific fleet segments (country, sector, year, gear), the number of days at sea in the most similar segment was used instead.

The nominal fishing effort (per fleet segment) was defined as :

$$Eff_{Nom} = P \times DAS \times R_{Act} \quad (2), \text{with}$$

- Eff_{Nom} the nominal effort (in kW*Days),
- P the total engine power (fleet),
- DAS the number of days at sea ,
- R_{act} the ratio of vessels in activity to the total capacity.

Data on the activity of the fleet was found only for a quarter of the world's countries (Figure S8), mainly European countries and dependencies, and rarely separated by sector, but by motorization. For the countries with enough data, the activity ratio of the fleet was extrapolated using gam, or given the country average if data was lacking. With a few exceptions[16], it was considered that the rest of the world fishing fleet had an average activity rate of 72%.

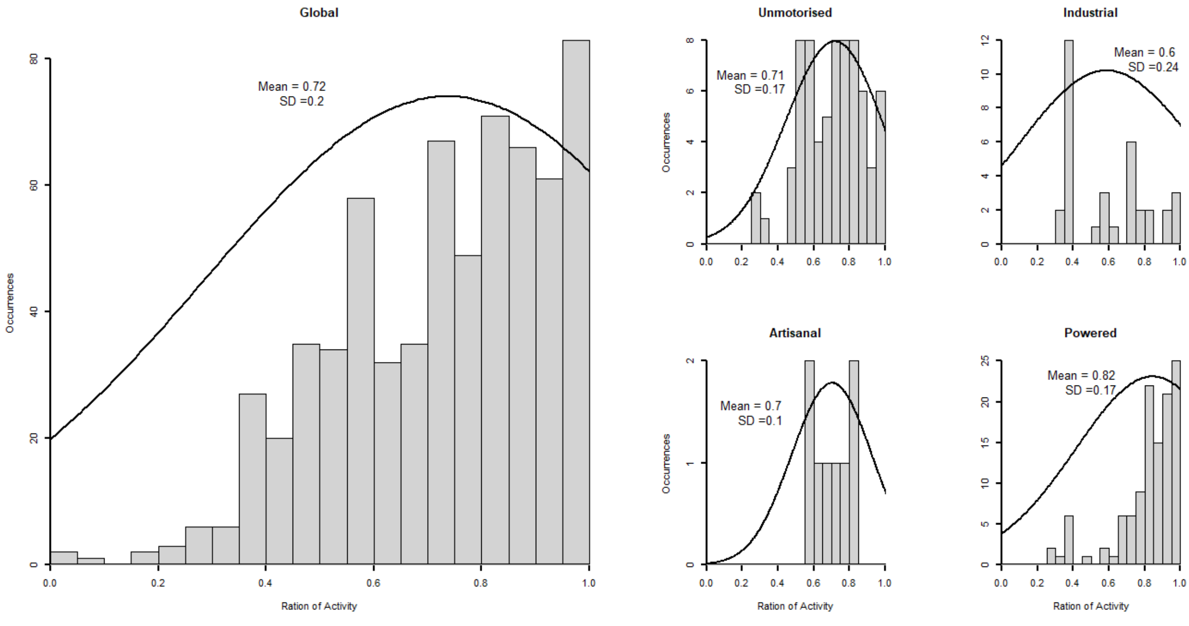

16 Fleet data for Chile, the Maldives and the industrial fleet of PNG and Oman were reported as active, therefore given a ratio of Activity of 1. Since most of the Ukrainian industrial fleet was located in Crimea (with the exception of one boat), the industrial effort of Ukraine was considered null from 2014 onwards[11]. The Iran-Iraq War effectively reduced the fishing activity of Iraq from 1980 to 1988 to zero, increasing to a quarter, third and half in the following year [12,13].

Figure S8. Activity of marine fishing vessels, globally (left) and by sector/type of motorisation (right). "Powered" corresponds to motorised fleets for which the ratio of activity is not given by sector. Each occurrence corresponds to a data point (year) for a country.

Association Catch – Nominal effort

The catch dataset[7] is comprised of events, each of them associating a fishing country, gear, sector to species/functional groups, total catch/landings and location (0.5 degree cell) per year. The fishing effort and capacity by country, year, sector, length of vessel and gear type were associated to the catch using gear equivalence between the 2 databases (Level 1, Figure 9). When the gear used in the effort did not match any of the gear associations in the catch database then broader gear families were used (e.g. nets encompassing gillnets, liftnets and seines, Level 2, Figure 9), considering that some errors are possible in the classification due to misnomers, translation of terms and difficulty to determine one specific gear from another (such as troll lines and longlines). Further incompatibility between gears in the effort database vs the catch database led to using sector or country-level data.

The industrial fleet of a country was considered to be capable of fishing worldwide, so any catch event was kept. Artisanal motorized were limited to catch events occurring in the EEZ of the fishing country, while unmotorized artisanal were constrained to 12nm from the coast (limiting the catch events to those occurring within 2 cells from the coast). The result is an association of each effort event (country, sector, gear, length category of vessel, year) to a series of possible catch events.

Mapping Effort

Each catch event – effort event association was prorated according to the total gross tonnage of each associated effort event (Figure 10), assuming that a vessel will try to maximize the efficiency of the trip by holding as much catch as the tonnage allows. Each effort event was then prorated on locations (0.5 degree cells) based on the sum of the associated catch.

Converting Days at sea to Fishing hours

While the mapped data treats considers days at sea (x kW) as the base unit for the effort, analysis of the impact of effort on resources and comparability analysis often requires fishing hours. The daily AIS data per vessel (each allocated a Maritime Mobile Service Identity number, MMSI) was downloaded from the Global Fishing Watch website (https://globalfishingwatch.org/), and converted from 0.1 to 0.5 degree cells by aggregation. Each cell was allocated a distance to the coastline, and the average number of hours fished per day at sea was plotted and fitted with averaging GAM models for each class of vessel length (Figure 11). As AIS data is incomplete for smaller vessels, the value for vessels 6-12m was used for vessels under 6m.

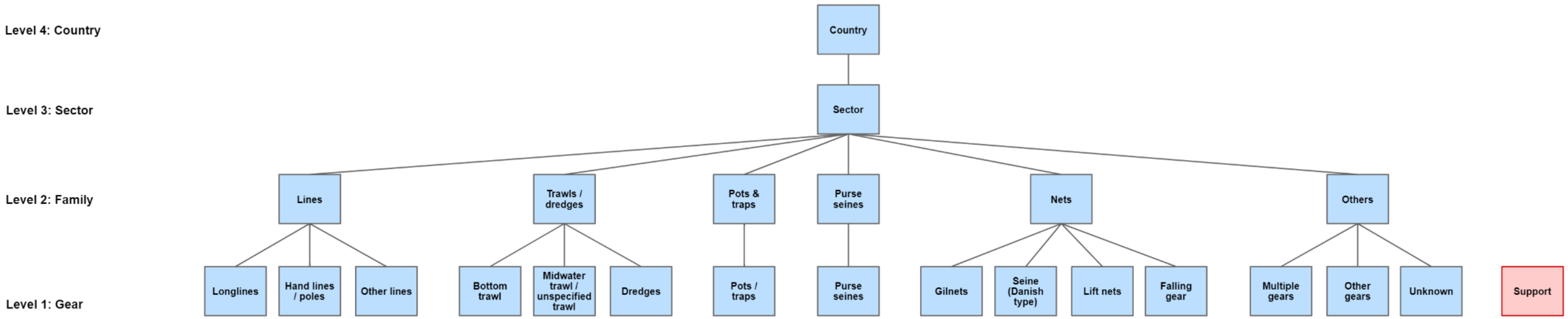

Figure S9. Hierarchy of gears and family used for association of the effort database with Catch. "Support" refers to support vessels, transport and motherboats, factories, which were not associated a catch value, as they do not actively participate in the fishing activity.

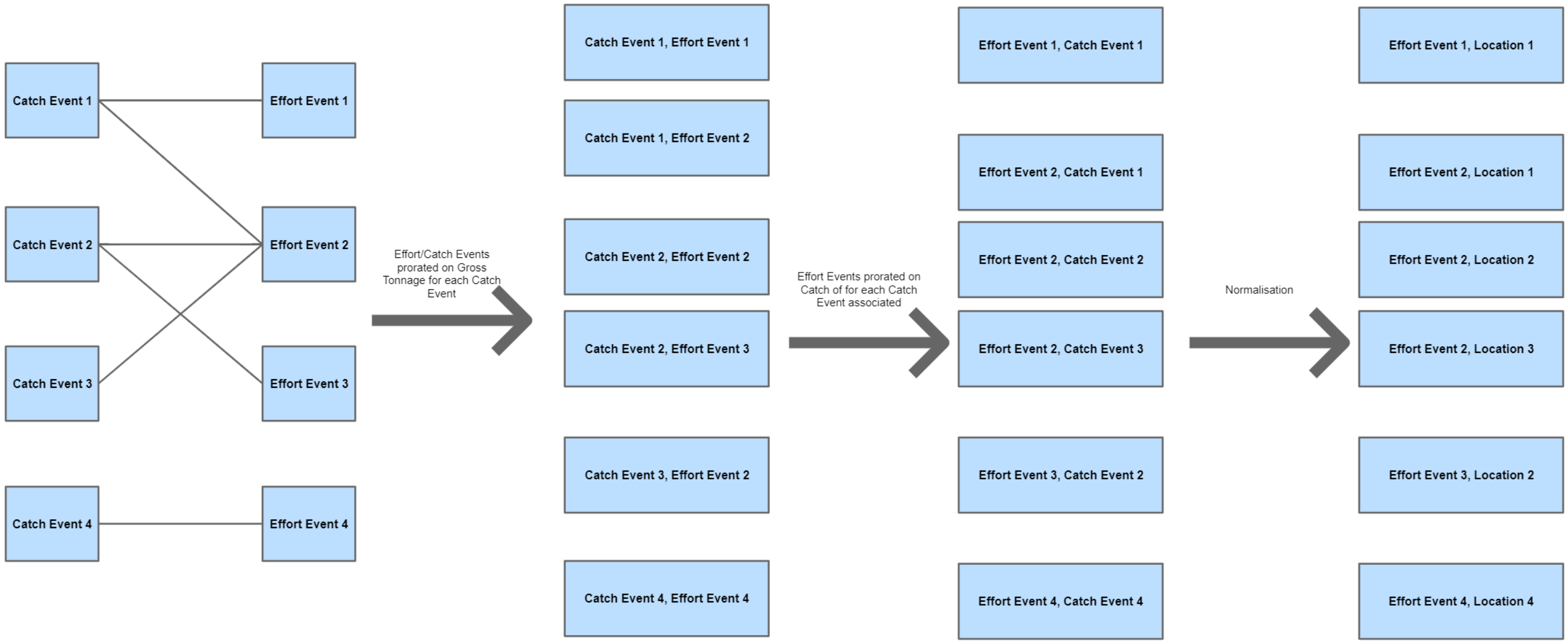

Figure S10. Prorates used in allocating a location to each effort event.

The number of fishing hours per day clearly shows steps and plateaux corresponding to the end of coastal waters (10-20km from the coast) and of EEZ (370km). A decrease in fishing hours past 2000km for the largest vessels can be attributed to a few vessels navigating across entire oceans.

The conversion factors between fishing hours and fishing days

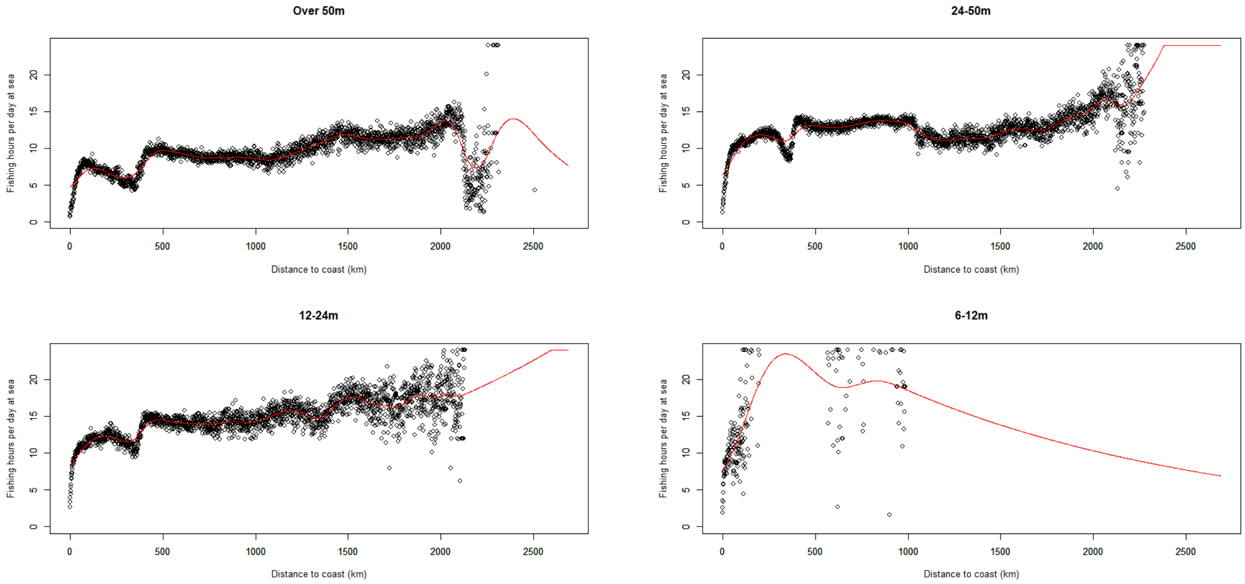

Figure S11. Average fishing hours per day at sea for different lengths of vessels, according to the distance to the nearest shore.

Technological creep and effective fishing effort.

Unlike fishing capacity, which refers to the actual number of vessels and/or their attributes, the effort relates to their activity, or capacity utilization, linking more closely to fishing mortality [9]. The link between nominal and effective effort is found in the technological creep (C), which is defined as *the fraction of the change in catchability which* ***does not*** *stem from changes in the engine power of the fleet or in the status of the stock (biomass-independent and engine-power independent creep).*

Catchability (q) is the fraction of the stock that is available to be fished (per unit effort) at any point in time, linking to the catch:

$$\widehat{\mathrm{Q}}Catch = \widehat{\mathrm{Q}}[q \cdot f \cdot B] \quad = \frac{q_2 \cdot f_2 \cdot B_2}{q_1 \cdot f_1 \cdot B_1} \qquad (3), \text{ where:}$$

- $\widehat{\mathrm{Q}}$ represents the ratio operator ($\widehat{\mathrm{Q}}x = \widehat{\mathrm{Q}}(x) = \frac{x_2}{x_1}$),
- f is the effort,
- B is the biomass of the stock

This definition[17] does not, however, capture the fact that the catchability depends on both human aspects (q_H , changes in gear, boat design, knowledge, …) and biological aspects (q_B , availability and density of biomass, behavior, changes in life history).

The 'human' catchability q_H is in fact the catchability linked to the creep in (3):

$$\widehat{\mathrm{Q}}\, q = \widehat{\mathrm{Q}}\, [q_H \times q_B] = C \times \widehat{\mathrm{Q}}\, q_B \quad (4)$$

Leading to:

$$\mathrm{C} = \widehat{\mathrm{Q}}q\big|_{kW,B\ Constant} = \widehat{\mathrm{Q}}\left(\frac{q}{q_B}\right) = \widehat{\mathrm{Q}}\left(\frac{Catch}{f \cdot B \cdot q_B}\right) = \widehat{\mathrm{Q}}\left(\frac{CPUE}{B \times q_B}\right) \quad (5)$$

The link between biomass-dependent catchability q_B and the biomass was explored with a logistic model:

$$q_{B,logistic} = \frac{a}{\exp^{-r\frac{B_i}{B_0}}} \quad (6), \text{ with}$$

- a, coefficients of the models (linked to maximum)
- B_i the stock biomass at time i, B_0 the pristine stock biomass
- And r the logistic growth rate.

Leading to:

$$C_{y_1,y_2,log} = \widehat{\mathrm{Q}}\left(\frac{Catch}{Effort \times B \times q_B}\right) = \frac{CPUE_2}{CPUE_1} \times \frac{B_1}{B_2} \times \exp^{-r\left(\frac{B_2}{B_0} - \frac{B_1}{B_0}\right)} \quad (7)$$

The nominal fishing effort was matched with the catch data by sector, gear and country, and with stock assessment [10] in order to calculate both the variation in CPUE and biomass per

[17] Strictly speaking, being a temporal (yearly) change, the variation should be written as $\widehat{\mathrm{Q}}(x) = \frac{x_2 - x_1}{x_1} = \frac{x_2}{x_1} - 1$. The "-1" is forgotten from the equation from that point onwards for simplicity.

year, gear (or family of gear) and country/region. Stock assessment was limited to stocks exploited mainly by one fishing country (excluding all massively transboundary stocks such as tunas).

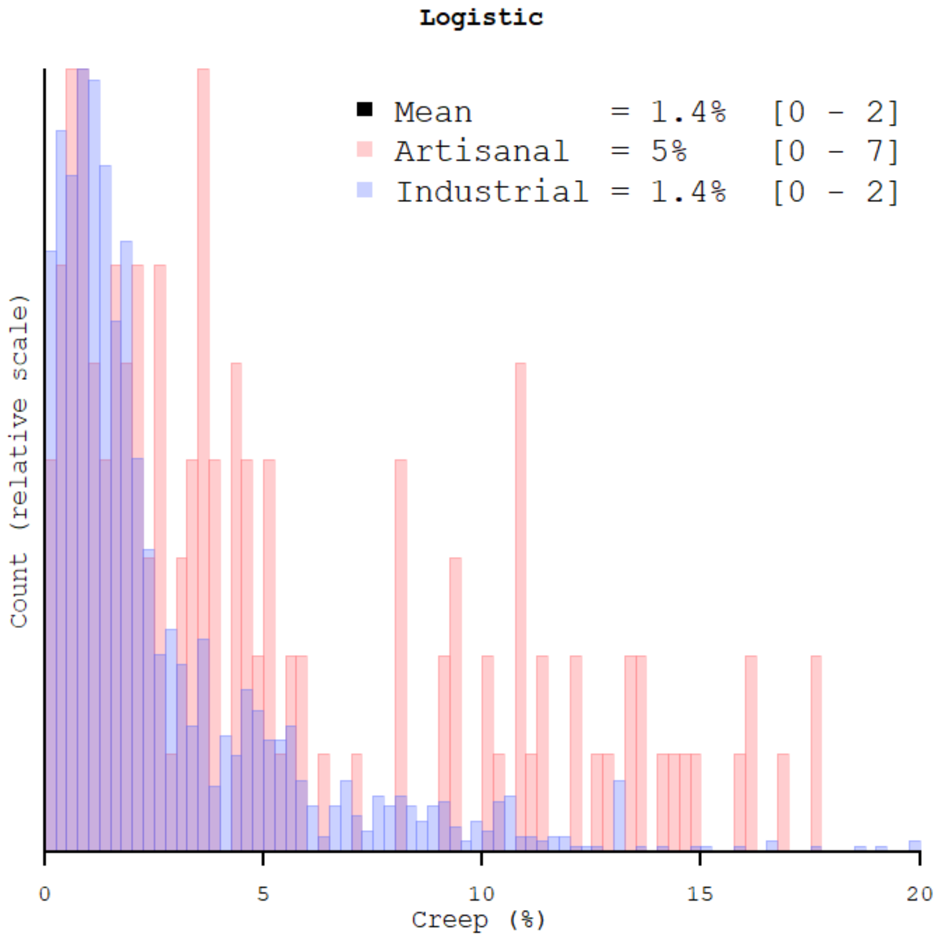

Figure S12. Creep, calculated with logistic biomass-dependent catchability, by sector. Each count corresponds to one year, country and gear type. Mean is calculated according to a beta distribution, with number in brackets corresponding to the highest 75% probability density interval.

Code Availability

Data analysis was carried using readily available (open source) R code and packages. Example code for effort mapping and to explore fishing capacity (R-shiny) is provided in https://github.com/Global-Fishing-Effort/RousseauEtAl2023.

The Code used to extrapolate and interpolate data using R supposes a dataframe “Data” of columns “Year” and “Number_of_Vessels” (for each country and sector):

```
library(gam)
```

```
Data$Predictor <- Data$Number_of_Vessels # if the number of vessels is the extrapolated data

Data$Predictor[Data$Predictor == 0] <- NA # Need to remove 0 for GAM to function.

k <-  min(10, length (round(unique(Data$Year[is.na(Data$Predictor)==F])/10) )-1  )

gam_model <- gam(Predictor ~ s(Year,k),  family=gaussian(link="log"), data= Data, se=TRUE)

Data$Predicted <- round( predict(gam_model, newdata=Data, type='response'))

Data$Predicted[is.na(Data$Number_of_Vessels)==F] <- Data$Predicted[ is.na( Data$Number_of_Vessels) == F]
```

Similar code is used for all time-series, including extrapolation of the number of vessels, the ratio of each vessel length/gears, average gross tonnage, average engine power.

Data Citations

Rousseau, Y., Blanchard, J., Novaglio, C., ~~Kirsty,~~ Pinnell, K., Tittensor, D., Watson, R., & Ye, Y. *Institute for Marine and Antarctic Studies, University of Tasmania*. DOI:10.25959/MNGY-0Q43 (2022)

References:

1. Rousseau, Y., Watson, R. A., Blanchard, J. L. & Fulton, E. A. Evolution of global marine fishing fleets and the response of fished resources. *Proceedings of the National Academy of Sciences* **116**, 12238–12243 (2019).

2. Rousseau, Y., Watson, R. A., Blanchard, J. L. & Fulton, E. A. Defining global artisanal fisheries. *Marine Policy* **108**, 103634 (2019).

3. Pauly, D. & Zeller, D. Sea around us, concepts, design and data. (2016).

4. Bell, J. D., Watson, R. A. & Ye, Y. Global fishing capacity and fishing effort from 1950 to 2012. *Fish and Fisheries* **18**, 489–505 (2017).

5. Hastie, T. *Package ' gam .'* (CRAN Library, 2020).

6. *Survey of safety at sea issues in Pacific Islands artisanal fisheries*. (Food and Agriculture Organization of the United Nations RAS/89/039 and United NAtion Development Programme TCP/RAS/0058, 1991).

7. Watson, R. A. A database of global marine commercial, small-scale, illegal and unreported fisheries catch 1950–2014. *Scientific Data* **4**, 170039 (2017).

8. Anticamara, J. A., Watson, R. A., Gelchu, A. & Pauly, D. Global fishing effort (1950-2010): Trends, gaps, and implications. *Fisheries Research* **107**, 131–136 (2011).

9. Ward, J. M., Kirkley, J. E., Metzner, R. & Pascoe, S. *Measuring and assessing capacity in fisheries - 1. Basic concepts and management options*. (Food and Agriculture Organization of the United Nations, 2004).

10. Ricard, D., Minto, C., Jensen, O. P. & Baum, J. K. Examining the knowledge base and status of commercially exploited marine species with the RAM Legacy Stock Assessment Database. *Fish and Fisheries* **13**, 380–398 (2012).

11. Shuvar. Ukrainian fishing industry: imports, consumption reduce to a third (Українська рибна галузь: імпорт, скорочення споживання втричі). (2017).

12. Ali, T. S., Mohamed, A. M. & Hussain, N. A. The status of Iraqi marine fisheries during 1990-1994. *Marina Mesopotamica* **13**, 129–147 (1998).

13. Mohamed, A. R. M., Ali, T. S. & Hussain, N. A. The physical oceanography and fisheries of Iraqi marine waters, Northwest Arabian Gulf. in *Proceedings on utilization of marine resources.* 47–56 (Islamic Educational, Scientific and Cultural Organization, 2005).
